# Low Psoas Lumbar Vertebral Index Is Associated with Mortality after Hip Fracture Surgery in Elderly Patients: A Retrospective Analysis

**DOI:** 10.3390/jpm11070673

**Published:** 2021-07-16

**Authors:** Ji-Hoon Sim, Soo-Ho Lee, Ji-Wan Kim, Won-Uk Koh, Hyung-Tae Kim, Young-Jin Ro, Ha-Jung Kim

**Affiliations:** 1 Department of Anesthesiology and Pain Medicine, Asan Medical Center, University of Ulsan College of Medicine, 88, Olympic-ro 43-gil, Songpa-gu, Seoul 05505, Korea; atlassjh@hanmail.net (J.-H.S.); koh9726@naver.com (W.-U.K.); ingwei2475@gmail.com (H.-T.K.); yjro@amc.seoul.kr (Y.-J.R.); 2 Department of Anesthesiology and Pain Medicine, College of Medicine, Catholic Kwandong University, International ST. Mary’s Hospital, Incheon 22711, Korea; rysooho84@gmail.com; 3 Department of Orthopaedic Surgery, Asan Medical Center, University of Ulsan College of Medicine, 88, Olympic-ro 43-gil, Songpa-gu, Seoul 05505, Korea; bakpaker@hanmail.net

**Keywords:** sarcopenia, elderly, psoas lumbar vertebra index, hip fracture, mortality

## Abstract

The psoas-to-lumbar index (PLVI) has been reported as a simple and easy way to measure central sarcopenia. However, only few studies have evaluated the association between PLVI and survival in surgical patients. This study evaluated the association between preoperative PLVI and mortality in elderly patients who underwent hip fracture surgery. We retrospectively analyzed 615 patients who underwent hip fracture surgery between January 2014 and December 2018. The median value of each PLVI was calculated according to sex, and the patients were categorized into two groups on the basis of the median value (low PLVI group vs. high PLVI group). Cox regression analysis was performed to evaluate the risk factors for 1 year and overall mortalities. The median values of PLVI were 0.62 and 0.50 in men and women, respectively. In the Cox regression analysis, low PLVI was significantly associated with higher 1 year (hazard ratio (HR): 1.87, 95% confidence interval (CI): 1.18–2.96, *p* = 0.008) and overall mortalities (HR: 1.51, 95% CI: 1.12–2.03, *p* = 0.006). Low PLVI was significantly associated with a higher mortality. Therefore, PLVI might be an independent predictor of mortality in elderly patients undergoing hip fracture surgery.

## 1. Introduction

The aging population is increasing worldwide [1,2]. Hip fracture surgeries are also on the rise with the aging population, with more than approximately 100 cases per 100,000 reported annually in Asian countries [3]. The number of hip joint surgeries will continue to increase in the future [4] and surpass six million by the year 2050 [5].

Age is a well-known risk factor for predicting surgical outcomes in elderly patients requiring hip fracture surgery [6,7]. Recently, not only the patient’s chronological ages but also their biological ages were shown to be important [8,9,10]. In particular, the concept of frailty is emerging as a useful predictor of a patient’s physiological reserve to recover from certain stressful events [11,12]. Frailty is considered an important factor influencing the morbidity and mortality of various diseases in patients of advanced ages, including many types of surgery [8,12,13,14,15,16,17]. Frailty is commonly described as slowness, weakness, weight loss, low activity, and fatigue [9,12]. Some recent studies have suggested several screening tools for measuring frailty [12,16,17,18]. However, these tools are usually dependent on patients’ ability to provide adequate medical history and their physical performance [12,16,17]. Therefore, application of these screening tools may be difficult in elderly patients with cognitive impairments or physical limitations, including hip fracture. Therefore, alternatively, central sarcopenia, measured using radiographs, has been proposed as a surrogate for frailty [18,19,20]. One of the simple and easy ways to define central sarcopenia is to determine the psoas-to-lumbar index (PLVI) [19]. The association between PLVI and surgical outcomes has been reported in cardiovascular procedures and thoracic surgery [19,21]; however, the effect of central sarcopenia in elderly patients requiring hip fracture surgery has not been fully established yet.

The objective of this study was to evaluate the association between radiographically determined PLVI and mortality in elderly patients undergoing hip fracture surgery and to assess whether PLVI can be a surrogate for central sarcopenia.

## 2. Materials and Methods

### 2.1. Study Design and Patients

**Figure 1:**
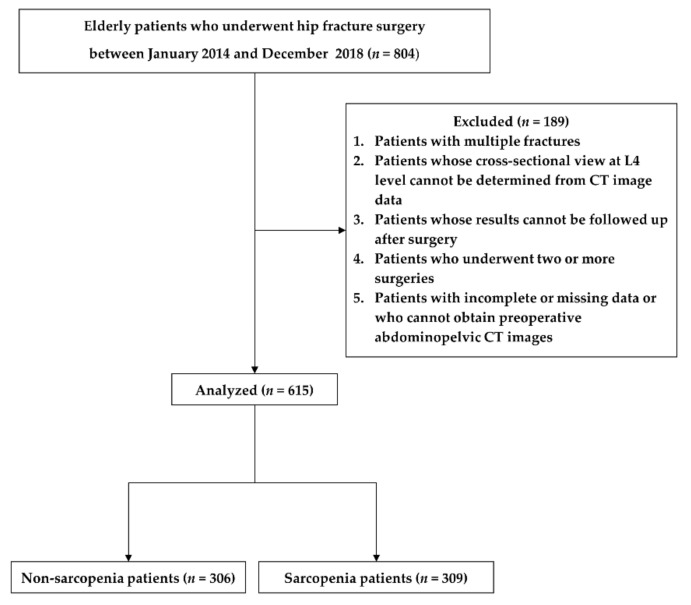
Study flowchart.

This retrospective study was approved by the Institutional Review Board of the Asan Medical Center (protocol code 2019-1100; August 2019), and the requirement for written informed consent was waived given the retrospective nature of the study. The data of patients who underwent hip fracture surgery such as arthroplasty or osteosynthesis from January 2014 to December 2018 were reviewed. Patients with multiple fractures, which were not simple fractures, in whom the cross-sectional view of the L4 level could not be determined using computed tomography (CT) or the cross-sectional area of the lumbar vertebrae or psoas could not be measured, whose results were not followed up after surgery, who underwent two or more surgeries, and with incomplete or missing data or whose preoperative CT images could not be obtained were excluded. Of the 804 enrolled patients who underwent hip fracture surgery, 189 were excluded on the basis of the study criteria. Thus, a total of 615 patients were enrolled in this study (Figure 1).

### 2.2. Measurements

In all patients, CT was performed according to the standard clinical protocol, and the CT image acquisition parameters were adjusted according to the patient’s body weight. The CT images were reviewed by two authors who were blinded to the patient information in the picture archiving and communication system (PACS).

**Figure 2:**
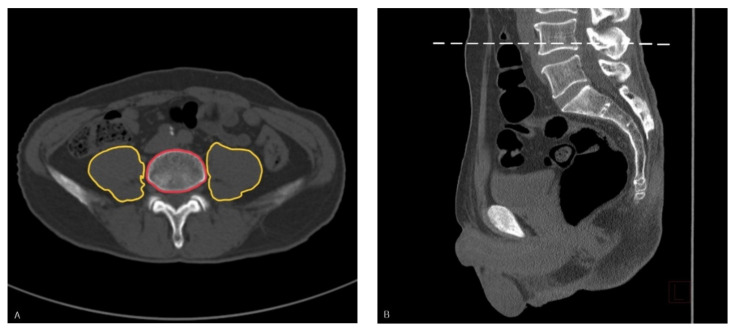
(**A**) Representative cross-sectional image. Measurement of the right and left psoas muscle areas (outlined in yellow) and vertebral body area (outlined in red) at the L4 level using abdominopelvic computed tomography images; (**B**) Representative sagittal image. The cross-sectional image was obtained at the level of the dashed line.

As an indicator of sarcopenia, the PLVI was used, a measure that has been established in similar studies [19,21]. PLVI is defined as the ratio of the mean area of the right and left psoas muscles to the sum of the areas of lumbar vertebrae. Axial CT sections at the level of the inferior endplate of the fourth lumbar vertebrae (L4) were selected, and the areas of the left and right psoas muscles, and L4 vertebral body were manually calculated using measurements in PACS (Figure 2). This method has been reported as a valid assessment of central sarcopenia and a facile manual measurement. The median value of each PLVI was calculated on the basis of sex, and the patients were categorized into two groups according to the median value (low PLVI group and high PLVI group).

### 2.3. Data Collection and Outcome Assessment

Demographic and perioperative variables of patients were obtained from the medical record system at our institution. The demographic variables included age, sex, body mass index (BMI), American Society of Anesthesiologists (ASA) classification, and comorbidities such as diabetes mellitus (DM), hypertension (HTN), cerebrovascular disease (CVA), cardiovascular disease (CVD), pulmonary disease, kidney disease, and cognitive impairment. Variables related to patients’ muscle mass included the left and right psoas muscle areas, L4 vertebrae areas, and PLVI. Preoperative laboratory values included those of hemoglobin (Hb), platelet, white blood cell (WBC), serum albumin, aspartate transaminase (AST), alanine transaminase (ALT), C-reactive protein (CRP), and serum creatinine. Intraoperative variables included operation type, operation timing, and anesthesia type. Data on postoperative hospital stay, postoperative delirium, 1 year mortality, and overall mortality were also collected.

The main outcome of this study was to investigate the association of PLVI with 1 year and overall mortalities in elderly patients with hip fracture surgery. Other risk factors for 1 year and overall mortalities were also analyzed as a secondary outcome.

### 2.4. Statistical Analyses

Data are described as means ± standard deviations, medians (interquartile ranges), or numbers (percentages), as appropriate. The chi-squared or Fisher’s exact test was used for categorical data, and the independent *t*-test or Mann–Whitney U-test was used for continuous data. The cumulative risks of 1 year and overall mortalities were estimated using the Kaplan–Meier method in the two groups stratified by PLVI. The Cox proportional hazard regression model was used to identify the risk factors for 1 year and overall mortalities. All variables with *p* < 0.1 in the univariate analysis were included in the multivariate analysis. A *p*-value < 0.05 was considered statistically significant, and two-tailed *p*-values were used. All data were analyzed using SPSS Statistics version 22.0 for Windows (IBM Corp., Armonk, NY, USA) and R version 3.1.2 (R Foundation for Statistical Computing, Vienna, Austria).

## 3. Results

Table 1 presents the baseline characteristics, perioperative variables, and surgical outcomes of the study population. The mean age and BMI of the patients were 80.73 years and 22.08 kg/m^2^, respectively, and the majority of the patients were women. Of the 615 patients, 188 (30.6%) had DM, 353 (57.4%) had HTN, 129 (21.0%) had CVA, and 191 (31.1%) had CVD. The median duration of hospital stay was 7.00 days. Most of the patients belonged to ASA classes 2 (59.7%) and 3 (40.3%), and four patients belonged to ASA class 4 (1.0%). The incidence of postoperative delirium was 25.6% (159/615). The 1 year and overall mortalities were 14.5% (89/615) and 32.7% (201/615), respectively (Table 1).

The median values of PLVI were 0.62 and 0.50 in men and women, respectively. Patients in the low PLVI group were older (*p* < 0.001), belonged to higher ASA classes (*p* = 0.018), and had lower BMI (*p* < 0.001) than patients in the high PLVI group. In addition, more cases of delayed operation were observed in the low PLVI group (*p* = 0.001). Intergroup differences were nonsignificant in the other study demographic variables.

With respect to laboratory variables, patients with sarcopenia had a higher CRP (*p* = 0.003) and lower hemoglobin (*p* < 0.001), WBC count (*p* = 0.002), albumin level (*p* < 0.001), and ALT (*p* = 0.013) (Table 1). Patients in the low PLVI group had higher 1 year (*p* < 0.001) and overall mortalities (*p* < 0.001) than patients in the high PLVI group. However, differences in hospital stay and postoperative delirium incidence between the two groups were not significant.

### Study Outcomes

**Figure 3:**
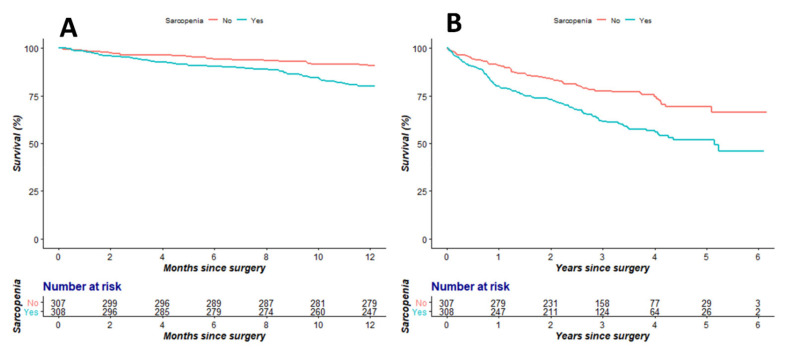
Kaplan–Meier curves for 1 year survival (**A**) and overall survival (**B**) according to sarcopenia defined by PLVI (log-rank test; *p* = 0.008 in 1 year survival, *p* = 0.006 in overall survival).

In the Cox regression analysis, low PLVI was significantly associated with 1 year (hazard ratio (HR): 1.87, 95% confidence interval (CI): 1.18–2.96, *p* = 0.008) (Table 2) and overall mortalities (HR: 1.51, 95% CI: 1.12–2.03, *p* = 0.006) (Table 3). Additionally, male sex (HR: 1.67, 95% CI: 1.08–2.59, *p* = 0.021) was an independent risk factor for 1 year mortality (Table 2), while age (HR: 1.04, 95% CI: 1.01–1.06, *p* = 0.001), male sex (HR: 1.67, 95% CI: 1.24–2.26, *p* = 0.001), ASA 3 or 4 (HR: 1.78, 95% CI: 1.30–2.45, *p* < 0.001), DM (HR: 1.43, 95% CI: 1.04–1.96, *p* = 0.026), CVA (HR: 1.41, 95% CI: 1.02–1.94, *p* = 0.037), hypoalbuminemia (HR: 1.72, 95% CI: 1.21–2.44, *p* = 0.002), and CRP (HR: 1.03, 95% CI: 1.00–1.07, *p* = 0.045) were significantly associated with the overall mortality (Table 3). Figure 3 shows the Kaplan–Meier curve of 1 year and overall mortalities according to the PLVI (log-rank test; *p* = 0.008 in 1 year mortality, *p* = 0.006 in overall mortality).

## 4. Discussion

This study demonstrated the association of preoperative PLVI with increased 1 year and overall mortalities in elderly patients requiring hip fracture surgery. According to this, PLVI can provide important information in predicting mortality in hip fracture surgery as a useful surrogate marker for central sarcopenia.

Recently, central sarcopenia has been reported as a surrogate for frailty [18,22]. Central sarcopenia can be evaluated by measuring the core muscle size, representatively psoas muscle [14,19,20,23]. The total psoas area (TPA) was reported to be associated with morbidity and long-term mortality in aortic valve replacement [20] and mortality in open abdominal aneurysm repair [13]. However, given that the TPA cutoff value can vary as a function of body size, adjustment for body size was required. Other studies have reported PLVI rather than TPA alone to reflect values on the basis of individual patients’ physical habits [19,24]. According to Ebbeling et al., central sarcopenia, defined as below the median value of PLVI, independently predicts morbidity in geriatric patients with trauma [19]. However, to the best of our knowledge, reports on the association between PLVI and surgical prognosis in patients with hip fractures are few. Therefore, this study is clinically meaningful as a major investigation on the association between PLVI and mortality.

According to our results, low PLVI, representing central sarcopenia, was associated with mortality after hip fracture surgery. Studies in various surgical populations have identified sarcopenia as an independent risk factor for morbidity and mortality [25,26,27]. According to a meta-analysis by Hajibandeh et al. [27], sarcopenia was associated with a higher risk of 30 day mortality, 1 year mortality, total complications, intensive care unit (ICU) care, prolonged ICU stay, and hospital stay in case of emergency abdominal surgery. Additionally, a higher risk of 30 day mortality has been reported in elective abdominal surgeries [27]. Frailty is considered to play a key role in these results, as sarcopenia is a major component of frailty and shares similarities in the etiology [28,29]. Corresponding to previous studies, patients with sarcopenia had a higher mortality in this study. Therefore, sarcopenia, defined by PLVI, can be used to predict mortality in hip fracture surgery.

Additionally, in this study, hypoalbuminemia was associated with mortality. Hypoalbuminemia is a well-known risk factor in patients undergoing surgical interventions [30,31,32]. Albumin is considered as a nutritional status indicator; therefore, hypoalbuminemia might serve as a marker for malnutrition [32,33,34]. The relationship between hypoalbuminemia and mortality can be explained by this pathophysiology [32]. Recently, a retrospective study conducted by Sim et al. of 971 patients undergoing hip fracture surgery [34] reported hypoalbuminemia to be associated with slower recovery and lower postoperative 6 month function and quality of life. Malnutrition is considered to be important in the development of sarcopenia and frailty, which is often overlapped in elderly patients [35]. However, in this study, hypoalbuminemia and low PLVI were both identified as independent risk factors for mortality after adjusting for confounding factors.

In our study, age was not a risk factor of 1 year mortality. The patient group included in our study had an average age of 80.73 years, and it is speculated that biological age may have played a more critical role than chronological age in this elderly patient group.

Between the two groups in this study, the low PLVI group had a higher CRP level and lower ALT, albumin, and hemoglobin levels. These results are consistent with those of previous studies evaluating biomarkers in frailty [36,37,38]. Inflammation and malnutrition are considered to play an important role in the pathophysiology of frailty; therefore, the variables reflecting these changes might be used as a biomarker for frailty [36,38]. However, these changes are affected by the natural aging process and are easily affected by the common comorbidities of old age [33,38,39]. Therefore, caution is required when interpreting laboratory test results, especially when the patient is under acute stress.

Using PLVI as an objective measure for sarcopenia and a surrogate for frailty has several clinical advantages, especially in patients undergoing hip fracture surgery. First, it is objective and easy to acquire compared with the other screening tools for frailty. Moreover, a special software package or extra training is not necessary for its determination. Therefore, PLVI can be easily used in various clinical settings. Second, assessment of the patient’s physical performance is not required, which is often not applicable in patients with hip fracture. Third, the need for additional examination is minimized, as cross-sectional imaging (CT or magnetic resonance imaging) studies are routinely performed in the early course of the diagnosis. Fourth, PLVI can be obtained without the patient’s cooperation or provision of medical history, which is often difficult in elderly patients with cognitive impairment. Considering these advantages, PLVI can be widely used in predicting mortality after hip fracture surgery in elderly patients. The management of patients with low PLVI requires more meticulous monitoring and care during the perioperative period to improve the clinical outcome.

Our study had some limitations. Firstly, given the retrospective design of the study, unaccounted confounding factors such as preoperative walking and physical performance may have triggered potential biases. However, given that our study purpose was to identify an objective indicator for patients with limited movement, these factors may not have had a significant effect on our results. Secondly, in this study, the patients were divided into two groups on the basis of the median PLVI values, and the group with a lower value was assumed to have sarcopenia. However, there is currently no unanimous agreement on the cutoff value in central sarcopenia. In addition, as the study patients were relatively older than those in the other studies and because there was an ethnic disparity, cutoff values from other studies were not used in this study. Although our cutoff point demonstrated positive results, it may not apply to other studies. Lastly, this was a single-center study with a limited number of patients and an ethnically homogeneous group comprising mostly South Korean patients. A multicenter study including a larger cohort and heterogeneous groups may be required.

## 5. Conclusions

In conclusion, low PLVI was significantly associated with higher mortality in elderly patients following hip fracture surgery. Therefore, PLVI might be a simple and objective way to assess central sarcopenia, which could be an independent predictor for mortality in elderly patients undergoing hip fracture surgery.

## Figures and Tables

**Table 1:** Baseline characteristics, perioperative variables, and surgical outcomes of the study population.

	Study Population			
	High PLVI	Low PLVI	Total	*p*-Value
	(*n* = 306)	(*n* = 309)	(*n* = 615)	
Demographic variables				
Age, year	79.24 ± 7.05	82.20 ± 7.97	80.73 ± 7.67	<0.001
Sex, male	87 (28.4)	88 (28.5)	175 (28.5)	0.990
BMI, kg/m^2^	23.09 ± 3.55	21.08 ± 3.85	22.08 ± 3.84	<0.001
DM	101 (33.0)	87 (28.2)	188 (30.6)	0.192
HTN	185 (60.5)	168 (54.4)	353 (57.4)	0.127
CVA	62 (20.3)	67 (21.7)	129 (21.0)	0.665
CVD	98 (32.0)	93 (30.1)	191 (31.1)	0.605
Pulmonary disease	64 (20.9)	74 (23.9)	138 (22.4)	0.367
Kidney disease	39 (12.7)	37 (12.0)	76 (12.4)	0.771
Cognitive impairment	36 (11.8)	39 (12.6)	75 (12.2)	0.745
ASA				0.018
2	192 (62.7)	169 (54.7)	367 (59.7)	
3	109 (35.6)	139 (45.0)	248 (40.3)	
4	5 (1.6)	1 (0.3)	6 (1.0)	
PLVI	0.65 ± 0.11	0.44 ± 0.09	0.55 ± 0.15	<0.001
Anesthesia type				
General	118 (38.6)	119 (38.5)	237 (38.5)	0.990
Spinal	188 (61.4)	190 (61.5)	378 (61.5)	
Operation timing				
Early (≤48 h)	136 (44.4)	98 (31.7)	234 (38.5)	0.001
Late (>48 h)	170 (55.6)	211 (68.3)	381 (62.0)	
Operation type				
Arthroplasty	138 (45.1)	117 (37.9)	255 (41.5)	0.069
Osteosynthesis	168 (54.9)	192 (62.1)	360 (58.5)	
Laboratory variables				
Hemoglobin, g/dL	11.45 ± 1.84	10.84 ± 1.92	11.20 ± 1.91	<0.001
Platelet, 10^9^/L	204.25 ± 74.28	211.69 ± 78.18	200.00 ± 76.29	0.177
WBC, 10^3^/uL	10.44 ± 3.82	9.67 ± 3.64	9.60 ± 3.75	0.002
CRP, mg/dL	1.99 ± 3.65	2.60 ± 3.89	0.640 ± 3.7824	0.003
Albumin, g/dL	3.44 ± 0.44	3.22 ± 0.52	3.400 ± 0.4933	<0.001
AST, IU/L	27.85 ± 22.03	24.54 ± 11.00	22.00 ± 17.45	0.072
ALT, IU/L	18.02 ± 11.61	16.13 ± 9.59	15.00 ± 10.67	0.013
Creatinine, mg/dL	1.13 ± 1.25	1.04 ± 1.10	0.78 ± 1.18	0.489
Surgical outcomes				
Hospital stay (days)	7.00 (5.00, 10.75)	7.00 (6.00, 10.00)	7.00 (5.00, 10.00)	0.672
Delirium	75 (24.5)	84 (27.2)	159 (25.9)	0.449
1 year mortality	28 (9.2)	61 (19.7)	89 (14.5)	<0.001
Overall mortality	75 (24.5)	126 (40.8)	201 (32.7)	<0.001

Values are expressed as means ± standard deviations, medians (interquartile ranges), or absolute numbers (percentages). BMI, body mass index; DM, diabetes mellitus; HTN, hypertension; CVD, cardiovascular disease; CVA, cerebrovascular disease; ASA, American Society of Anesthesiologists; CRIF, closed reduction and internal fixation; PLVI, psoas-to-lumbar vertebral index; WBC, white blood cell; CRP, C-reactive protein; AST, aspartate aminotransferase; ALT, alanine aminotransferase.

**Table 2:** Cox regression analyses of 1 year mortality.

	Univariate			Multivariable		
	HR	95% CI	*p*-Value	HR	95% CI	*p*-Value
Low PLVI	2.26	1.45–3.54	<0.001	1.87	1.18–2.96	0.008
Age	1.02	1.00–1.05	0.074	1.01	0.98–1.04	0.354
Sex (male)	2.02	1.33–3.08	0.001	1.67	1.08–2.59	0.021
ASA						
2	1.00			1.00		
3/4	2.21	1.45–3.37	<0.001	1.47	0.92–2.35	0.103
BMI	0.96	0.91–1.02	0.182			
DM	1.79	1.18–2.73	0.006	1.57	0.99–2.50	0.057
HTN	1.42	0.92–2.20	0.111	1.15	0.72–1.85	0.561
CVD	1.48	0.97–2.27	0.070	1.09	0.69–1.73	0.698
CVA	1.47	0.92–2.34	0.108			
Cognitive impairment	1.64	0.95–2.81	0.074	1.24	0.70–2.17	0.462
Hypoalbuminemia (albumin < 3.5 g/dL)	2.61	1.62–4.20	<0.001	1.65	0.99–2.72	0.053
Anemia (Hb < 12 g/dL)	2.49	1.41–4.41	0.002	1.68	0.93–3.04	0.088
Operation timing						
Early (≤48 h)	1.00			1.00		
Late (>48 h)	1.96	1.21–3.18	0.006	1.30	0.78–2.17	0.308

Values are expressed as means ± standard deviations, medians (interquartile ranges), or absolute numbers (percentages). HR, hazard ratio; CI; confidence interval; PLVI, psoas-to-lumbar index; BMI, body mass index; DM, diabetes mellitus; HTN, hypertension; CVD, cardiovascular disease; CVA, cerebrovascular disease; ASA, American Society of Anesthesiologists.

**Table 3:** Cox regression analyses of the overall mortality.

	Univariate			Multivariable		
	HR	95% CI	*p*-Value	HR	95% CI	*p*-Value
Low PLVI	1.84	1.38–2.45	<0.001	1.51	1.12–2.03	0.006
Age	1.05	1.03–1.06	<0.001	1.04	1.01–1.06	0.001
Sex (male)	2.16	1.63–2.86	<0.001	1.67	1.24–2.26	0.001
ASA						
2	1.00			1.00		
3 or 4	2.74	2.06, 3.64	<0.001	1.78	1.30–2.45	<0.001
BMI	0.92	0.89–0.96	<0.001			
DM	1.67	1.26–2.22	<0.001	1.43	1.04–1.96	0.026
HTN	1.45	1.09–1.94	0.011	1.21	0.89–1.66	0.226
CVD	1.51	1.14–2.02	0.004	1.05	0.76–1.43	0.782
CVA	1.83	1.35–2.48	<0.001	1.41	1.02–1.94	0.037
Pulmonary disease	1.56	1.14–2.12	0.005	0.99	0.70–1.41	0.971
Kidney disease	1.69	1.17–2.44	0.006	1.04	0.70–1.53	0.850
Cognitive impairment	1.91	1.33–2.73	<0.001	1.21	0.89–1.66	0.226
Anesthesia type						
General	1.00			1.00		
Spinal	1.06	0.79–1.41	0.713	1.04	0.77–1.41	0.802
Hypoalbuminemia (albumin < 3.5 g/dL)	2.73	2.01–3.73	<0.001	1.72	1.21–2.44	0.002
Anemia (Hb < 12 g/dL)	2.16	1.52–3.05	<0.001	1.42	0.97–2.07	0.069
CRP	1.09	1.06–1.12	<0.001	1.03	1.00–1.07	0.045
Operation timing						
Early (≤48 h)	1.00			1.00		
Late (>48 h)	1.59	1.18–2.14	0.003	0.93	0.66–1.30	0.652
Operation type						
Arthroplasty	1.00			1.00		
Osteosynthesis	0.86	0.65–1.13	0.280	0.76	0.57–1.03	0.075

Values are expressed as means ± standard deviations, medians (interquartile ranges), or absolute numbers (percentages). HR, hazard ratio; CI; confidence interval; PLVI, psoas-to-lumbar index; BMI, body mass index; DM, diabetes mellitus; HTN, hypertension; CVD, cardiovascular disease; CVA, cerebrovascular disease; ASA, American Society of Anesthesiologists.

## Data Availability

The data presented in this study are available on request from the corresponding author. The data are not publicly available due to conditions of the ethics committee of our university.

## References

[1] Hoogendijk E.O., Afilalo J., Ensrud K.E., Kowal P., Onder G., Fried L.P. (2019). Frailty: Implications for clinical practice and public health. Lancet.

[2] Dent E., Martin F.C., Bergman H., Woo J., Romero-Ortuno R., Walston J.D. (2019). Management of frailty: Opportunities, challenges, and future directions. Lancet.

[3] Lin K.B., Yang N.P., Lee Y.H., Chan C.L., Wu C.H., Chen H.C., Chang N.T. (2018). The incidence and factors of hip fractures and subsequent morbidity in Taiwan: An 11-Year Population-Based cohort study. PLoS ONE.

[4] Lewiecki E.M., Wright N.C., Curtis J.R., Siris E., Gagel R.F., Saag K.G., Singer A.J., Steven P.M., Adler R.A. (2018). Hip fracture trends in the United States, 2002 to 2015. Osteoporos. Int..

[5] Kannus P., Parkkari J., Sievänen H., Heinonen A., Vuori I., Järvinen M. (1996). Epidemiology of hip fractures. Bone.

[6] Huette P., Abou-Arab O., Djebara A.E., Terrasi B., Beyls C., Guinot P.G., Havet E., Dupont H., Lorne E., Ntouba A. (2020). Risk factors and mortality of patients undergoing hip fracture surgery: A One-Year Follow-Up study. Sci. Rep..

[7] Kim S.D., Park S.J., Lee D.H., Jee D.L. (2013). Risk factors of morbidity and mortality following hip fracture surgery. Korean J. Anesthesiol..

[8] Richards S.J.G., Frizelle F.A., Geddes J.A., Eglinton T.W., Hampton M.B. (2018). Frailty in surgical patients. Int. J. Colorectal Dis..

[9] Walston J., Hadley E.C., Ferrucci L., Guralnik J.M., Newman A.B., Studenski S.A., Ershler W.B., Harris T., Fried L.P. (2006). Research agenda for frailty in older adults: Toward a better understanding of physiology and etiology: Summary from the American Geriatrics Society/National Institute on Aging Research Conference on Frailty in Older Adults. J. Am. Geriatr. Soc..

[10] Carneiro J.A., Cardoso R.R., Durães M.S., Guedes M.C.A., Santos F.L., Costa F.M.D., Caldeira A.P. (2017). Frailty in the elderly: Prevalence and associated factors. Rev. Bras. Enferm..

[11] Campbell A.J., Buchner D.M. (1997). Unstable disability and the fluctuations of frailty. Age Ageing.

[12] Fried L.P., Tangen C.M., Walston J., Newman A.B., Hirsch C., Gottdiener J., Seeman T., Tracy R., Kop W.J., Burke G. (2001). Frailty in older adults: Evidence for a phenotype. J. Gerontol. Ser. A Biol. Sci. Med. Sci..

[13] Lee J.S.-J., He K., Harbaugh C.M., Schaubel D.E., Sonnenday C.J., Wang S.C., Englesbe M.J., Eliason J.L. (2011). Michigan Analytic Morphomics Group. Frailty, core muscle size, and mortality in patients undergoing open abdominal aortic aneurysm repair. J. Vasc. Surg..

[14] Moskven E., Bourassa-Moreau E., Charest-Morin R., Flexman A., Street J. (2018). The impact of frailty and sarcopenia on postoperative outcomes in adult spine surgery. A systematic review of the literature. Spine J..

[15] James L.A., Levin M.A., Lin H.M., Deiner S.G. (2019). Association of Preoperative Frailty with Intraoperative Hemodynamic Instability and Postoperative Mortality. Anesth. Analg..

[16] Bautista M.A.C., Malhotra R. (2018). Identification and Measurement of Frailty: A Scoping Review of Published Research from Singapore. Ann. Acad. Med. Singap..

[17] Walston J., Buta B., Xue Q.L. (2018). Frailty Screening and Interventions: Considerations for Clinical Practice. Clin. Geriatr. Med..

[18] Thompson M.Q., Yu S., Tucker G.R., Adams R.J., Cesari M., Theou O., Visvanathan R. (2021). Frailty and sarcopenia in combination are more predictive of mortality than either condition alone. Maturitas.

[19] Ebbeling L., Grabo D.J., Shashaty M., Dua R., Sonnad S.S., Sims C.A., Pascual J.L., Schwab C.W., Holena D.N. (2014). Psoas: Lumbar vertebra index: Central sarcopenia independently predicts morbidity in elderly trauma patients. Eur. J. Trauma Emerg. Surg..

[20] Hawkins R.B., Mehaffey J.H., Charles E.J., Kern J.A., Lim D.S., Teman N.R., Ailawadi G. (2018). Psoas Muscle Size Predicts Risk-Adjusted Outcomes After Surgical Aortic Valve Replacement. Ann. Thorac. Surg..

[21] Mitchell P.M., Collinge C.A., O’Neill D.E., Bible J.E., Mir H.R. (2018). Sarcopenia is Predictive of 1-Year Mortality After Acetabular Fractures in Elderly Patients. J. Orthop. Trauma.

[22] Jones K.I., Doleman B., Scott S., Lund J.N., Williams J.P. (2015). Simple psoas Cross-Sectional area measurement is a quick and easy method to assess sarcopenia and predicts major surgical complications. Colorectal Dis..

[23] Okamura H., Kimura N., Tanno K., Mieno M., Matsumoto H., Yamaguchi A., Adachi H. (2019). The impact of preoperative sarcopenia, defined based on psoas muscle area, on Long-Term outcomes of heart valve surery. J. Thorac. Cardiovasc. Surg..

[24] Swanson S., Patterson R.B. (2015). The correlation between the psoas muscle/vertebral body ratio and the severity of peripheral artery disease. Ann. Vasc. Surg..

[25] Newton D.H., Kim C., Lee N., Wolfe L., Pfeifer J., Amendola M. (2018). Sarcopenia predicts poor Long-Term survival in patients undergoing endovascular aortic aneurysm repair. J. Vasc. Surg..

[26] Liu P., Hao Q., Hai S., Wang H., Cao L., Dong B. (2017). Sarcopenia as a predictor of All-Cause mortality among community-dwelling older people: A systematic review and Meta-Analysis. Maturitas.

[27] Hajibandeh S., Hajibandeh S., Jarvis R., Bhogal T., Dalmia S. (2019). Meta-Analysis of the effect of sarcopenia in predicting postoperative mortality in emergency and elective abdominal surgery. Surgeon.

[28] Nascimento C.M., Ingles M., Salvador-Pascual A., Cominetti M.R., Gomez-Cabrera M.C., Viña J. (2019). Sarcopenia, frailty and their prevention by exercise. Free Radic. Biol. Med..

[29] Brown N.A., Zenilman M.E. (2010). The impact of frailty in the elderly on the outcome of surgery in the aged. Adv. Surg..

[30] Abraham A., Burrows S., Abraham N.J., Mandal B. (2020). Modified frailty index and hypoalbuminemia as predictors of adverse outcomes in older adults presenting to acute general surgical unit. Rev. Esp. Geriatr. Gerontol..

[31] Ryan S., Politzer C., Fletcher A., Bolognesi M., Seyler T. (2018). Preoperative Hypoalbuminemia Predicts Poor Short-Term Outcomes for Hip Fracture Surgery. Orthopedics.

[32] Kim S., McClave S.A., Martindale R.G., Miller K.R., Hurt R.T. (2017). Hypoalbuminemia and Clinical Outcomes: What is the Mechanism behind the Relationship?. Am. Surg..

[33] Loftus T.J., Brown M.P., Slish J.H., Rosenthal M.D. (2019). Serum Levels of Prealbumin and Albumin for Preoperative Risk Stratification. Nutr. Clin. Pract..

[34] Sim S.D., Sim Y.E., Tay K., Howe T.S., Png M.A., Chang C.C.P., Abdullah H.R., Koh J.S.B. (2021). Preoperative hypoalbuminemia: Poor functional outcomes and quality of life after hip fracture surgery. Bone.

[35] Cruz-Jentoft A.J., Kiesswetter E., Drey M., Sieber C.C. (2017). Nutrition, frailty, and sarcopenia. Aging Clin. Exp. Res..

[36] Kane A.E., Sinclair D.A. (2019). Frailty biomarkers in humans and rodents: Current approaches and future advances. Mech. Ageing Dev..

[37] Nam J.S., Kim W.J., An S.M., Choi D.K., Chin J.H., Lee E.H., Choi I.C. (2019). Age-Dependent relationship between preoperative serum aminotransferase and mortality after cardiovascular surgery. Aging.

[38] Al Saedi A., Feehan J., Phu S., Duque G. (2019). Current and emerging biomarkers of frailty in the elderly. Clin. Interv. Aging.

[39] Zou H.B., Yan X.L., Dong W.X., Yu D.Y., Zhang F.M., Zhou L.P., Shen Z.L., Cai G.J., Zhuang C.L., Yu Z. (2021). Sarcopenia is a predictive factor of poor quality of life and prognosis in patients after radical gastrectomy. Eur. J. Surg. Oncol..

